# FDM 3D Printing and Soil-Burial-Degradation Behaviors of Residue of Astragalus Particles/Thermoplastic Starch/Poly(lactic acid) Biocomposites

**DOI:** 10.3390/polym15102382

**Published:** 2023-05-19

**Authors:** Zhibing Ni, Jianan Shi, Mengya Li, Wen Lei, Wangwang Yu

**Affiliations:** 1 School of Transportation Engineering, Nanjing Vocational University of Industry Technology, Nanjing 210023, China; 2 College of Science, Nanjing Forestry University, Nanjing 210037, China; 3 School of Mechanical Engineering, Nanjing Vocational University of Industry Technology, Nanjing 210023, China

**Keywords:** astragalus residue powder, thermoplastic starch, poly(lactic acid), biocomposite, fused deposition modeling, soil burial, mechanical property, thermal property, degradation behavior

## Abstract

Astragalus residue powder (ARP)/thermoplastic starch (TPS)/poly(lactic acid) (PLA) biocomposites were prepared by fused-deposition modeling (FDM) 3D-printing technology for the first time in this paper, and certain physico-mechanical properties and soil-burial-biodegradation behaviors of the biocomposites were investigated. The results showed that after raising the dosage of ARP, the tensile and flexural strengths, the elongation at break and the thermal stability of the sample decreased, while the tensile and flexural moduli increased; after raising the dosage of TPS, the tensile and flexural strengths, the elongation at break and the thermal stability all decreased. Among all of the samples, sample C—which was composed of 11 wt.% ARP, 10 wt.% TPS and 79 wt.% PLA—was the cheapest and also the most easily degraded in water. The soil-degradation-behavior analysis of sample C showed that, after being buried in soil, the surfaces of the samples became grey at first, then darkened, after which the smooth surfaces became rough and certain components were found to detach from the samples. After soil burial for 180 days, there was weight loss of 21.40%, and the flexural strength and modulus, as well as the storage modulus, reduced from 82.1 MPa, 11,922.16 MPa and 2395.3 MPa to 47.6 MPa, 6653.92 MPa and 1476.5 MPa, respectively. Soil burial had little effect on the glass transition, cold crystallization or melting temperatures, while it reduced the crystallinity of the samples. It is concluded that the FDM 3D-printed ARP/TPS/PLA biocomposites are easy to degrade in soil conditions. This study developed a new kind of thoroughly degradable biocomposite for FDM 3D printing.

## 1. Introduction

Three-dimensional (3D) printing, also known as additive manufacturing, makes it possible to design a product with accurate numerical values of the dimensions through a computer graphic program and create 3D physical objects with exact dimensions in a relatively short time [1,2,3]. As one of the 3D-printing technologies, fused-deposition modeling (FDM) 3D printing is gaining much attention at present. In this process, the filaments prepared from thermoplastic materials are extruded through a nozzle under some designed operating conditions and successively deposited in a melted/softened state on the print bed to form the end items [4,5].

Up to now, certain traditional polymers and their composites have been applied as feedstocks for FDM 3D printing. For example, Rahmatabadi et al. [6] investigated the FDM 3D printing of PLA-TPU compounds with different component ratios, finding that the glass transition temperatures remained almost the same for various samples; furthermore, raising the proportion of PLA in the compound would increase the loss modulus, strength and fracture toughness, while simultaneously decreasing the storage modulus and formability of the samples. Rahmatabadi et al. [7] successfully FDM-3D-printed polyvinyl chloride (PVC) samples using different printing parameters for the first time. Among all of the concerned parameters, raster angle and printing velocity had the greatest effects on the mechanical properties of the samples, whereas the nozzle diameter and layer thickness had little effect; the maximum tensile strength reached 88.55 MPa, which showed the superiority of 3D-printed PVC mechanical properties compared to other commercial filaments. 

With the increasing awareness of environmental protection among people and the need for sustainable development of society, the research and application of biodegradable materials is becoming more and more important. For these reasons, biodegradable polymers, such as poly(lactic acid) (PLA), poly(butylene adipate-co-terephthalate) (PBAT) [8], butadiene styrene copolymer (PBS) [9], polycaprolactone (PCL) [10] and polyhydroxybutyrate (PHB) [11], have been chosen as the raw materials for FDM 3D printing. Among these, PLA has gained the most acceptance. PLA, synthesized from agricultural resources such as corn and tapioca, is biocompatible, compostable, recyclable, gas permeable and degradable by hydrolysis and enzymatic action. PLA is quite suitable for FDM printing because of its low melting point, low thermal expansion coefficient and lack of a pungent smell when being processed. However, its unit price is much more expensive than that of petroleum-based plastics, such as polyethylene and polypropylene. In addition, it exhibits a longer degradation time. Therefore, there is an urgent need to reduce the cost of PLA and improve its ability to degrade. The incorporation of natural fibers into the resin has proved to be an effective method to solve the aforementioned problems. As a result of this, a variety of natural fibers have been introduced into PLA for 3D printing. For example, Zhang et al. [12] printed poplar powder/PLA composites utilizing lubricant (TPW604) and polyolefin elastomer (POE) as a flexibilizer. They found that the lubricant improved the fluidity but reduced the impact strength of 3D-printing materials. The POE could improve the fluidity and toughness of the printed parts, and a higher content of POE resulted in better properties in a certain range. Aumnate et al. [13] extracted kenaf cellulose fibers (KFs) from locally grown kenaf plants, then treated them with tetraethyl orthosilicate, and prepared KFs/PLA biocomposite materials, using polyethylene glycol as a plasticizer. They found that the melt viscosities of the biocomposites increased as the fibers were loaded, but significantly decreased with the addition of plasticizers. The prototypes made from these materials could be applied in sustainable textiles and apparel, personalized prostheses and certain medical devices. Jaya et al. [14] 3D printed a continuous pineapple-leaf-fiber-reinforced PLA composite and investigated the properties of the specimens for the first time. They found that it took the same time to print the composites as to PLA, the tensile strength of the composite was increased by the application of the continuous pineapple leaf fiber, while its elongation at break was lower than that of the pure PLA part. Delphine et al. [15] produced bamboo-fiber-reinforced PLA filaments for FDM, the modulus of the filament was influenced by the length over diameter ratio of the compounded fibers, and the stiffness of the long bamboo-fiber-reinforced PLA filament could be increased by 215%. Guen et al. [16] compared the properties of the rice husk/PLA and wood/PLA filaments for FDM. The two biomasses had different effects on the rheological behavior while having similar effects on the mechanical properties of the 3D-printed samples. The complex viscosity of the compound could be increased by wood powder while being conversely decreased by rice husk powder. The mechanical properties were predominantly affected by the deposition direction.

Astragalus is a typical Chinese traditional medicinal crop; it is often cooked to extract certain bioactive polysaccharides for medical purposes. After being cooked, however, its residue, as one kind of natural fiber, is rarely re-utilized, which not only pollutes the environment but also leads to resource waste. We have conducted previous research on the 3D printing of astragalus residue powder (ARP)/PLA biocomposite. In our previous works, using ARP/PLA as the raw material for FDM 3D printing was proven to be feasible [17].

As a cheap, biodegradable, excellent renewable and easily available polysaccharide, thermoplastic starch (TPS) has been regarded as the optimal additive for blending with certain degradable polymers such as PLA, PBS and PCL. In recent years, many endeavors have been made on these blends for FDM 3D printing. Agnieszka et al. [18] developed a biodegradable and compostable TPS/PLA composite filament for FDM and investigated the properties of the filament. The incorporation of TPS not only reduces the cost of the granulate but also results in a considerable improvement in hydrophilicity and susceptibility to hydrolytic degradation. The compostability of the composite could also be enhanced in contrast to that of commercial PLA printouts. The printability for FDM and the properties of TPS/polycaprolactone composites were investigated by Zhao et al. [19], who found that the samples had the best performance in the FDM process with a starch ratio of 9 ph at 80~90 °C. The low printing temperature made it possible to introduce some bioactive components to produce antibacterial and biocompatible materials for FDM. Ju et al. [20] prepared a TPS/PLA/PBAT composite for FDM 3D printing, the filament had successful printability, and the samples could be printed accurately. Meanwhile, they found that the mechanical properties of the samples could be improved significantly when the chain extender ADR4468 was used.

Based on the above discussions, it was learned that both ARP and TPS could be used to form composites with PLA for FDM 3D printing, both the printed ARP/PLA and TPS/PLA samples had adequate properties and a much lower cost than the pure PLA. To our understanding, however, there have been no reports in the literature regarding the application of an ARP/TPS/PLA biocomposite in FDM printing.

In this work, we fabricated ecofriendly PLA composites loaded with TPS and ARP by using the FDM 3D-printing technique. The effects of the dosages of TPS and ARP on the mechanical and thermal properties of the composites, as well as their mass changes when immersed in water, were first investigated. Next, a specific ARP/TPS/PLA biocomposite was buried in soil and the biodegradation behavior of the samples was studied.

## 2. Experimental

### 2.1. Materials and Reagents

PLA (American Nature Works Co., 3052D, Minnetonka, MN, USA) in pellet form was purchased from Shanghai Xingyun International Trade Co. Ltd., China (Shanghai, China); TPS, food grade, was obtained from Shandong Hengren Industry and Trade Co., Ltd., China (Tengzhou, China); ARP, which was passed through an 120-mesh sieve, was made in our lab.

### 2.2. Preparation of FDM Filaments

Based on our previous work, 11 wt.% was the best proportion for ARP to add into PLA for the FDM 3D printing of ARP/PLA pieces when the cost and quality of the filament were taken into consideration simultaneously [21]. In this paper, the maximum proportion of ARP in the composite was thus controlled within 11 wt.%.

The biocomposite samples under investigation in this paper were PLA with different ARP and TPS compositions as listed in Table 1 infused into the base polymer. In each sample, 8 wt.% glycerol of the total mass of PLA, TPS and ARP was added as the plasticizing agent to lower the brittleness of the composite and guarantee the smooth production of the filament and the subsequently printed specimen.

**Figure 1:**
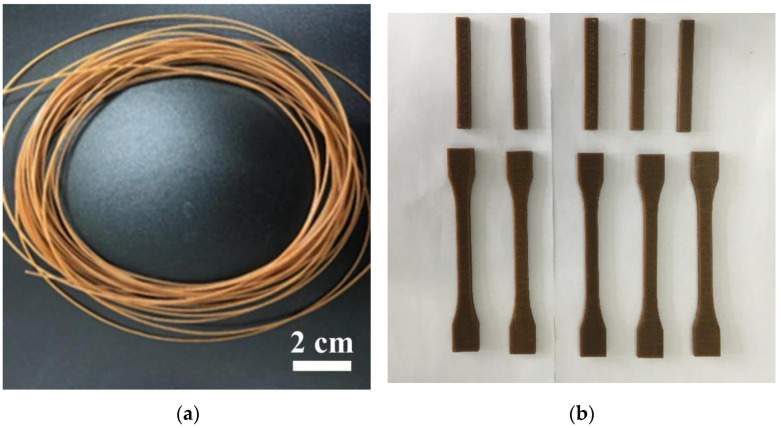
Physical images of the filaments and printed ARP/TPS/PLA biocomposite samples: (**a**) filaments; (**b**) printed parts.

The polymer composites using dried PLA, TPS and ARP as raw materials were first compounded with a twin screw extruder (SHJ-20, Nanjing Giant Machinery Co. Ltd., Nanjing, China) at 20 rpm and 130~160 °C, and the extrudate was granulated to make pellets; then, the filaments for FDM printing as illustrated in Figure 1a were prepared using a twin-screw extruder (KS-HXY, Kunshan Huanxinyang Electrical Equipment Co. Ltd., Suzhou, China) at 20 rpm and 170~190 °C.

### 2.3. Composite Preparation by FDM

Samples were printed with filaments prepared with the formulations listed in Table 1 on a desktop-level 3D printer (MOSHU S10, Hangzhou Shining 3D Technology Co. Ltd., Hangzhou, China) fitted with a 0.4-mm die nozzle. The printing parameters were chosen according to those for the FDM 3D printing of ARP/PLA biocomposite samples [21] and listed in Table 2.

The physical images of the printed samples are shown in Figure 1b.

### 2.4. Soil Degradation

The printed ARP/TPS/PLA parts were buried in soil collected in a 30 × 20 × 20 cm^3^ paper carton, then the soil degradation test was conducted at room temperature for up to 180 days. The soil moisture during the test was controlled between 17.5% and 21.5%. At each time interval (30, 60, 90 or 180 days after burial), the parts were taken out from the soil, cleaned with water and dried thoroughly in a hot-air oven. The changes of the parts in weight, surface color, mechanical properties, thermal stability, melting and cold crystallization behavior, as well as the thermal dynamic mechanic properties, were investigated.

### 2.5. Testing and Characterization

#### 2.5.1. Mechanical Testing

The samples were printed into dog-bone shapes for the tensile tests and rectangular shapes for the flexural tests, then the mechanical tests were performed using a universal testing machine (E44.304, MTS Industrial Systems (China) Co. Ltd., Shenzhen, China) with a load cell of 20 kN. The tensile and flexural tests were carried out according to the ASTM D 638 and ASTM D 790 standard testing methods with a crosshead speed of 10 mm/min and 5 mm/min, respectively.

#### 2.5.2. Thermal Stability

The thermal stability of the samples under a nitrogen atmosphere was analyzed using a TG 209F1 thermogravimetric analyzer (NETZSCH-Gerätebau GmbH, Selb, Germany). The experiments were performed on approximately 8-mg samples from 20 °C to 550 °C at a 20 K/min heating rate to investigate changes in the initial decomposition temperature (T_i_), thermal stability, and char residue of the samples.

#### 2.5.3. Mass Change in Water

The printed dog-bone-shaped samples were chosen for water immersion experiments. The samples were dried at 80 °C for 8 h, cooled in a desiccator, and then immediately massed to the nearest 0.0001 g. Thereafter, the samples were submerged in distilled water at room temperature, removed from the water after 7 d, gently blotted with tissue paper to remove excess water from their surfaces and immediately massed to the nearest 0.0001 g again. The percentage of mass change in water was calculated by weight variation between the samples immersed in water and the dry samples using the following formula:

(1) $$wu\left(\%\right)=\frac{w_{t}-w_{0}}{w_{0}}\times 100\%$$

where wu is the mass change rate, $w_{0}$ is the mass recorded before immersion and $w_{t}$ is the mass recorded after immersion.

#### 2.5.4. Weight Loss in Soil

The samples were taken out of the soil at every time interval, cleaned and then weighed. The weight loss rate (WL) of each sample was calculated by the weight variation between the samples before and after soil burial using the following formula:

(2) $$wl(\%)=\frac{w_{0}-w_{t}}{w_{0}}\times 100\%$$

where wl is the weight loss rate, $w_{0}$ is the mass recorded before soil burial and $w_{t}$ is the mass recorded after soil burial.

#### 2.5.5. Morphological Study (SEM)

The flexural fracture surfaces of the samples were first sputter-coated with a thin layer of gold to avoid any electrostatic charge and poor resolution during the scanning examination, and the SEM was operated at an accelerating voltage of 3 kV to image the samples at ×1000 magnification using a Hitachi SU 8010 field-emission scanning electron microscope (Hitachi Corporation, Tokyo, Japan).

#### 2.5.6. Melting and Crystallization Behavior

The melting and crystallization behavior was investigated using differential scanning calorimetry (DSC). Measurements were performed using a DSC214 (NETZSCH-Gerätebau GmbH, Selb, Germany), under a nitrogen flow of 20 mL/min. Samples of approximately 5~10 mg were cut from the 3D-printed samples and sealed in aluminum pans. The samples were heated from the ambient temperature to 220 °C at a ramp rate of 10 °C/min, and held in an isothermal state for 5 min, then cooled down to room temperature at a rate of 10 °C/min and subsequently reheated to 220 °C at a rate of 10 °C. The enthalpies of melting (∆*H_m_*) and cold crystallization ($∆H_{cc}$) were evaluated using the NETZSCH analysis software by integrating the areas of the melting and cold crystallization peaks. The $T_{g}$, $T_{m}$ and $T_{CC}$ values were taken from the second heating curves. The crystallization percentage of each piece was obtained via the following equation:

(3) $$x_{c}=\frac{\left|\Delta H_{m}-\Delta H_{cc}\right|}{\omega \Delta H^{*}}\times 100\%$$

where ω is the mass fraction of PLA in the sample, $\Delta H_{m}$ is the melting enthalpy, $\Delta H_{cc}$ is the cold crystallization enthalpy and $\Delta H^{*}$ = 93.6 J/g is the melting enthalpy of 100% crystalline PLA [22].

#### 2.5.7. Thermal Dynamic Mechanic Testing 

A dynamic mechanical analyzer (DMA 242C, Netzsch, Bavaria, Germany) was employed to investigate the dynamic mechanical properties of the FDM-3D-printed ARP/TPS/PLA parts at different soil burial durations. The test was carried out under a nitrogen atmosphere and at a sinusoidal frequency of 3.33 Hz, over a temperature range of 30~120 °C at a heating rate of 5 °C/min. During the test, the sample was held by a dual cantilever fixture.

## 3. Results and Discussion

### 3.1. Effects of Compositions on Properties of the Biocomposites

#### 3.1.1. Mechanical Properties

**Figure 2:**
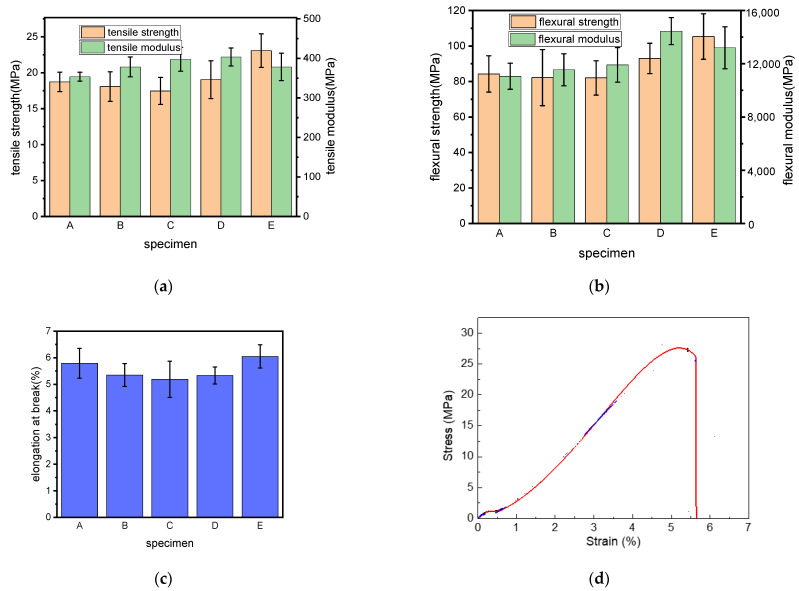
Mechanical properties of ARP/TPS/PLA biocomposite samples: (**a**) tensile strength and modulus, (**b**) flexural strength and modulus, (**c**) elongation at break, (**d**) a typical stress–strain curve of sample E during the tensile test.

The results of the tensile and bending tests conducted on the printed parts are presented in Figure 2. As the ARP loading increased (samples A, B and C), the values of the tensile and flexural moduli of the composites increased, while the corresponding strengths decreased slightly. For sample A, the tensile strength, the flexural strength, the tensile modulus and the flexural modulus were 18.73 MPa, 84.28 MPa, 353.61 MPa and 11,066.99 MPa, respectively; the corresponding strengths for sample C decreased to 17.46 MPa and 82.06 MPa, while the moduli increased to 397.48 MPa and 11,922.16 MPa, accordingly. The decrease in strength with the loading of the filler has been observed for several other natural fiber/polymer composites, such as peanut husk/poly(butylene adipate-co-terephthalate) (PBAT) [23], wood flour/PBAT [24] and sisal fiber/polypropylene [25]; this was attributed to the higher number of voids with higher filler content, as well as the poor dispersion of the hydrophilic natural fiber in the hydrophobic polymer matrix. According to the mixing rule, however, the modulus of the composite material was decided by the contribution of each component, the modulus of ARP/TPS/PLA was thus gradually increased when more ARP was used because of its greater modulus as a natural fiber than that of PLA. When the mechanical properties of samples C, D and E were taken into consideration, it was found that both the tensile and flexural strengths decreased with the increased substitution of PLA by TPS, and the elongation at break also decreased gradually, indicating that the incorporation of TPS would reduce the strengths of ARP/PLA composites and make the composite more fragile; thus, the content of TPS in the ARP/TPS/PLA composites should be controlled within a reasonable range. As an example, Figure 2d illustrates the stress–strain curve of sample E during the tensile test, a yield point appeared on the curve, meaning that this sample would show ductile fracture but not obviously. As reported, a complex thermal and mechanical history would be induced on the printed part during the FDM process; whether the yield point would appear on the stress–strain curve of the sample or not during the tensile test was directly decided by the free volume portion and its size distribution [26]. When ARP was incorporated into PLA, the free volume portion and its size distribution in the matrix changed. Meanwhile, the tensile strength of ARP is greater than the strength and adhesion between them; with the loading of the specimen, the fibers change direction in the loading direction [27], and thus behave as a ductile break, different from the commonly recognized fragile break of pure PLA [28].

#### 3.1.2. Thermal Properties

**Figure 3:**
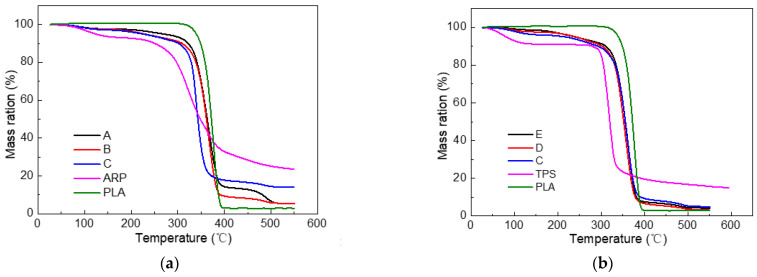
The mass loss curve of different specimens: (**a**) PLA, ARP and biocomposites with different ARP content; (**b**) PLA, TPS and biocomposites with different TPS content.

Figure 3 presents the TGA curves of the composites and their raw materials. It shows that all of the composites exhibited similar thermal degradation curves under identical conditions. Figure 3 further indicates that all of the composites were more easily thermally degraded than PLA while being much more difficult to thermally degrade than TPS and ARP. The main decomposition happened between 330 °C and 370 °C. Table 3 lists the characteristic thermal degradation parameters of the thermogravimetric curves of various composites, such as the initial temperature at which the sample began to decompose (T_i_) and the temperature at the maximum decomposition rate (T_p_). For sample A, T_i_ was 335.8 °C and T_p_ was 370.3 °C. For samples B and C, the T_i_ values decreased to 334.1 °C and 326.4 °C. Meanwhile, the T_p_ values decreased to 363.8 °C and 354.9 °C, respectively, indicating that the thermal stability became poorer with the replacement of more PLA by ARP. This is in accordance with the conclusions reported in many studies that the incorporation of natural fiber would worsen the thermal stability of PLA [28,29,30]. The reason is that the thermal stability of ARP is much poorer than PLA, as evidenced in Figure 3a.

Where samples C, D and E were concerned, it was found that upon increasing the percentage of TPS in the biocomposite, the sample would become more thermally unstable, which was mainly caused by the easier decomposition of TPS than PLA as depicted in Figure 3b.

#### 3.1.3. Mass Change in Water

**Figure 4:**
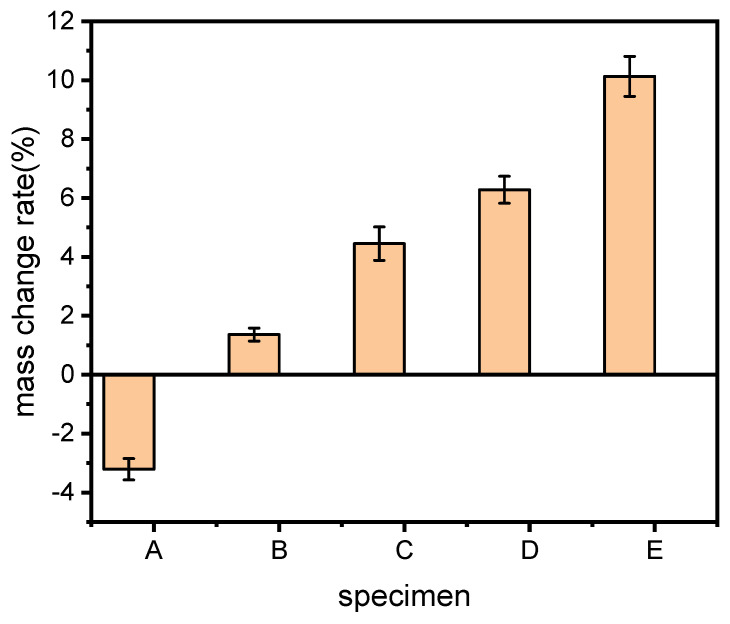
Mass change rate of the samples.

The mass change rate in water of each sample was demonstrated in Figure 4. It can be found that the mass of sample A was actually reduced after immersion in water for 7 d, the mass change rate was a negative value. When the dosage of ARP was increased, i.e., for samples B and C, the mass change rate of the samples increased gradually to positive values. When the content of ARP was kept constant in the specimen, the mass change rate was monotonically reduced with the increase in the amount of TPS. When the ARP/TPS/PLA sample was immersed in water, TPS was easily dissolved, resulting in mass reduction in the sample. The calculated mass change rate was accordingly decreased even though all of the components may absorb some water, and the sample containing more TPS lost its mass more heavily; this is why the total mass of the biocomposite sample reduced gradually with the increase in TPS. As one kind of natural fiber, ARP has many hydrophilic groups such as hydroxyl on its molecular structure, which combine with water molecules once they contact water. For the ARP/TPS/PLA composite with little ARP, the mass loss caused by the dissolvement of TPS held the dominant position. As a result, the total mass decreased, and the calculated mass change rate by Equation (1) showed a negative value. When more ARP was used, the mass of water absorbed exceeded that of TPS dissolved; consequently, the total mass change of the sample became positive; thus, sample C had a much greater positive mass change rate than sample B. With the invasion of water molecules, the internal structure of the biocomposites may be expanded and finally destroyed. The dissolvement of TPS and the damage to the internal structure of the biocomposite illustrated that sample C should be the easiest to degrade in wet conditions among all of the samples.

To sum up, increasing the dosage of ARP would reduce the strengths and thermal stability but improve the moduli of ARP/TPS/PLA biocomposites, while increasing the dosage of TPS would reduce the strengths and thermal stability and enhance the brittleness of ARP/TPS/PLA biocomposites. Even so, the mechanical and thermal properties of all of the samples could still meet the requirement for application. Among all of the samples, sample C was the easiest to degrade in wet conditions.

As a biodegradable composite, its cost must be taken into consideration for its wider application, in addition to possessing an excellent comprehensive performance. For ARP/TPS/PLA, the prices of each raw material differ greatly from one another, the prices of PLA and TPS are about 4500 USD/t and 500 USD/t in China, respectively. ARP is not available on the market as a kind of natural fiber; its price shall be about 80 USD/t when referring to the price of wood flour. The material cost of sample C will thus be about 3613.8 USD/t, 11.86% and 9.00% lower than those of samples A and E, respectively.

Taking into account the above factors comprehensively, sample C has adequate mechanical properties and thermal stability, though a little poorer than the other samples. Meanwhile, it is the most easily degraded in wet conditions and has a low cost; it was thus chosen as a research object in the following section, wherein the degradation behavior of sample C under soil burial was investigated.

### 3.2. Biodegradation Behavior of the Biocomposites

#### 3.2.1. Visual Appearance

**Figure 5:**
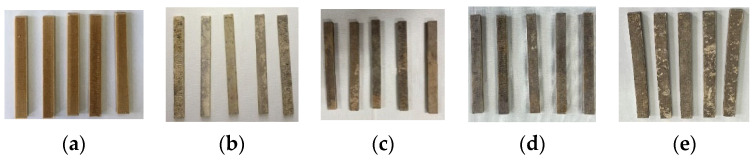
Visual appearances of ARP/TPS/PLA samples at different soil-burial days: (**a**) 0; (**b**) 30; (**c**) 60; (**d**) 90; (**e**) 180.

Figure 5 demonstrates the visual appearances of the printed samples after soil burial for different periods. All of the unburied samples had smooth and uniformly colored surfaces; after soil burial for 30 days, all the surfaces became gray with some black spots; with the continuation of soil burial, however, the surfaces of the samples became darkened and the surface color became more and more severely uneven. Meanwhile, certain components were found to detach from the samples, leaving some micro holes on the bodies. All the phenomena mentioned showed that degradation happened to the samples, and this degradation became more serious when the samples were buried for a longer time.

#### 3.2.2. Weight Loss

**Figure 6:**
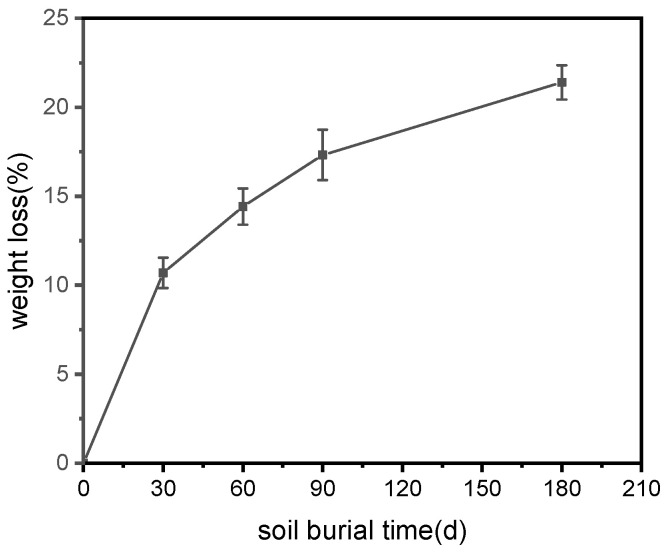
Weight losses of ARP/TPS/PLA samples with soil-burial days.

Figure 6 shows the percentage weight change as a function of soil-burial time for ARP/TPS/PLA composites. It was found that the sample lost its weight obviously with time: after being buried in soil for 180 days, the weight loss was 21.40%, indicating that the extension of soil-burial time would promote the biodegradation of ARP/TPS/PLA, which was consistent with the results from the visual appearance observation. Comparing the weight with that of pure PLA [21], it was found that ARP and TPS greatly accelerated the degradation of PLA.

#### 3.2.3. Prediction of Flexural Properties

**Figure 7:**
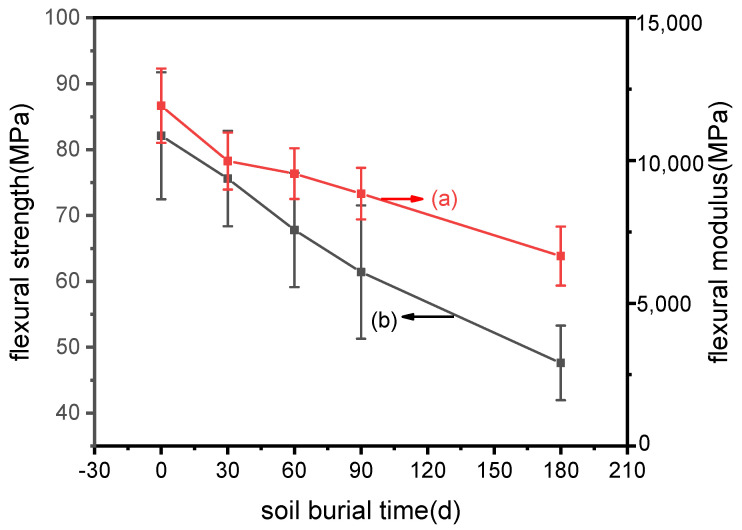
Bending properties’ changes of ARP/TPS/PLA samples with soil burial days: (**a**) flexural modulus: (**b**) flexural strength.

The effects of soil burial on the strength and modulus of ARP/TPS/PLA composites were investigated by bending tests. Figure 7 shows the trends of the properties with the soil-burial time. The flexural properties indicated that both the strength and modulus of ARP/TPS/PLA reduced gradually and almost linearly with time: after soil burial for 180 days, the flexural strength and modulus decreased to 47.6 MPa and 6653.92 MPa, reduced dramatically by 42.02% and 44.19% from those before soil burial. The fitting equations of the flexural strength and modulus could be expressed as FS = 80.69 − 0.19t, and FM = 11329.76 − 26.96t, respectively. Here, FS and FM represent the flexural strength and modulus, respectively, and t represents the soil burial days. It was derived from these two equations that the samples were speculated to lose their flexural strength and modulus thoroughly after soil burial for 376 d and 480 d, respectively. Although these were the results in the extreme cases predicted from the models, it could be expected that the samples would degrade greatly within 500 d, indicating that ARP/TPS/PLA was an easily biodegradable composite. 

#### 3.2.4. Cross-Sectional Morphologies

**Figure 8:**
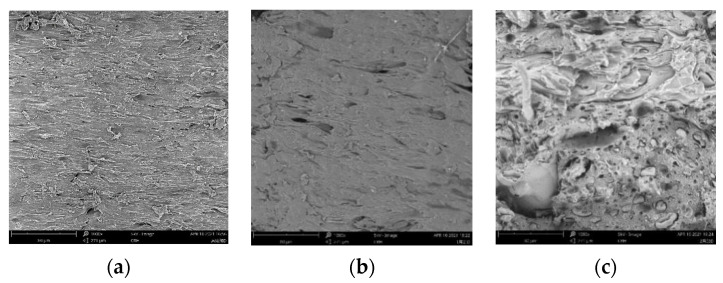

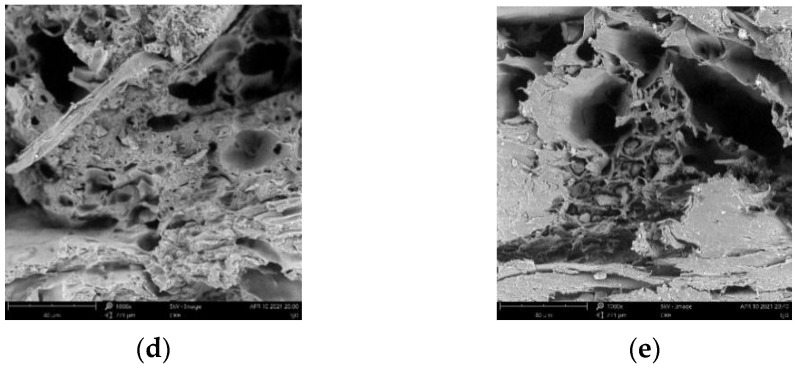
Cross-section morphology of ARP/TPS/PLA at different soil burial days: (**a**) 0; (**b**) 30; (**c**) 60; (**d**) 90; (**e**) 180.

The SEM images at ×1000 magnification after fracture obtained from 3D printing materials at different soil burial stages were shown in Figure 8. The pristine ARP/TPS/PLA had a relatively smooth fracture surface, showing that both ARP and TPS were enclosed well by PLA; however, in the case of the soil-buried samples, the fracture surface became much rougher and more cracks or holes appeared with the prolonging of the soil burial, and this phenomena was the most obvious for the sample after being buried in soil for 180 days. The existence of the cracks or holes made it easier for water to be absorbed by and transported in the sample; consequently, the internal structure of the sample would be destroyed more easily and the composite would degrade more heavily.

#### 3.2.5. Thermogravimetric Analysis

**Figure 9:**
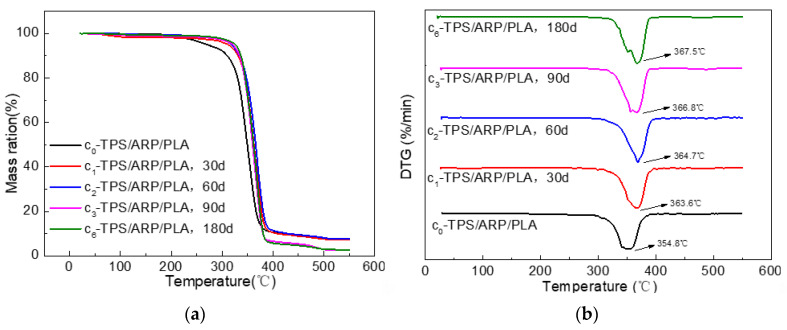
(**a**) TGA curves and (**b**) their derivatives of ARP/TPS/PLA biocomposites.

The thermal decomposition of the samples at different soil burial stages was investigated under a nitrogen atmosphere. Figure 9a,b illustrates the TGA and DTG thermograms during the decomposition of the printed samples in the temperature range of 20~550 °C, respectively.

The derivative of TGA (DTG) curves in Figure 8b and the corresponding testing values in Table 4 indicate that the main decomposition of each sample fell into the range between 300 °C and 400 °C. Both the initial decomposition and the maximum degradation temperatures of the samples increased with the extension of soil-burial time. After being buried in soil for 180 d, the T_i_ and T_p_ values of the samples were 340.0 °C and 367.5 °C, increased by 10.6 °C and 12.7 °C from those of the unburied samples, respectively, meaning that the soil burial improved the thermal stability of the samples. This trend was similar with that of rice straw powder/PLA biocomposites [22].

The possible reasons for the improved thermal stability of ARP/TPS/PLA biocomposites might come from three aspects. Firstly, PLA, as a polymer, was composed of crystal and amorphous domains; after being buried in soil, the amorphous domains would be destroyed by microorganisms, leaving the more thermally stable crystal domains, PLA itself may behave as more thermally stable in this situation. Secondly, TPS, as a polysaccharide, was easily degraded by microorganisms in the soil; the reduced amount of TPS in the soil was helpful for the improvement in the thermal stability of the samples because of its much poorer thermal stability than PLA as shown in Figure 3. Thirdly, ARP, as one kind of natural fiber, may easily rot in soil by itself; meanwhile, it was also much more thermally unstable than PLA and the biocomposite as shown in Figure 3. With the proceeding of soil burial, more and more ARP rotted and its content in the biocomposite reduced further and further, leading to the enhanced thermal stability of the whole sample.

#### 3.2.6. DSC Thermal Analysis

**Figure 10:**
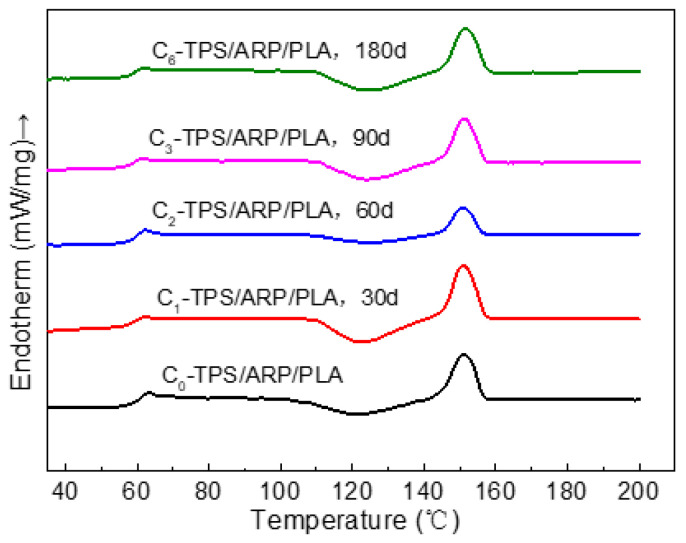
The secondary heating curves of the samples at different degradation periods.

The glass transition temperature (T_g_), melting point (T_m_) and cold crystallization temperature (T_cc_) are the important parameters for the composites. These parameters were investigated by a two-step heating cycle using DSC operating under a nitrogen atmosphere. The secondary heating curves in the DSC traces of the samples at different soil burial stages are shown in Figure 10, and the corresponding properties of the biocomposites are presented in Table 5. The T_g_, T_cc_ and T_m_ were 62.7 °C, 121.2 °C and 150.9 °C, respectively, for unburied ARP/TPS/PLA. The T_g_ decreased, while both the T_cc_ and T_m_ increased with the prolonging of the soil burial; however, these changes were not statistically significant. However, the degree of crystallinity showed a small decrease with the prolonging of the soil burial. This may be due to the following two reasons, one is that ARP, TPS and the amorphous areas in PLA were gradually destroyed during degradation, and a bigger free volume appeared in the matrix; as a result, the chain segment motion ability was enhanced, leading to a gradually reduced glassy transition temperature; meanwhile, the cold crystallization and melting would happen at higher temperatures. The other is that ARP, as a natural fiber, could act as a nucleating agent for the polymer matrix. When it was gradually degraded in the soil, the degree of crystallinity would be reduced accordingly. 

#### 3.2.7. Thermo-Dynamic Mechanical Properties

**Figure 11:**
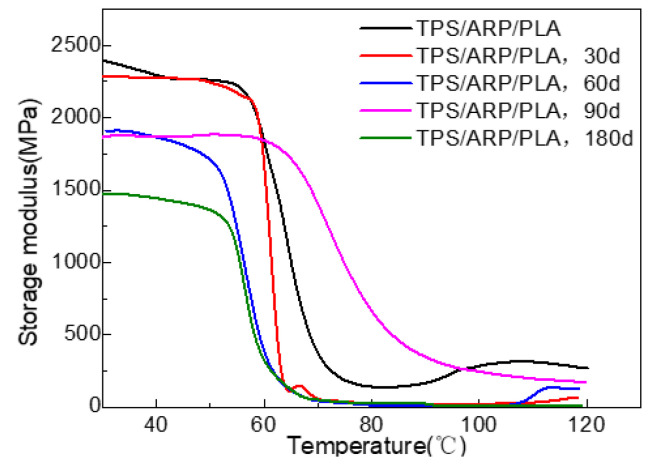
Storage-modulus curves of the samples at different degradation periods.

Figure 11 shows the DMA results of the soil-buried samples. The storage modulus (E′) (30 °C) shown in the figure was 2395.3 MPa, 2284.9 MPa, 1921.2 MPa, 1866.4 MPa and 1476.5 MPa for the samples buried in soil for 0, 30, 60, 90 and 180 days, respectively. Generally, the extension of soil-burial time decreased the E′ value gradually, and the value was significantly decreased by 38.4% after 180 d of soil burial. The existence of the hydrophilic natural fiber and starch made the sample more easily invaded and destroyed by water and microorganisms, accelerating the breaking of the molecular chains; as a result, the storage modulus of the sample was greatly reduced.

## 4. Conclusions

This study first investigated the effect of the dosages of ARP and TPS on the mechanical properties, thermal stability and mass changes in water of FDM 3D-printed ARP/TPS/PLA biocomposite samples, then studied the effects of soil-burial duration on the degradation behavior of the printed samples containing 11 wt.% ARP, 10 wt.% TPS and 79 wt.% PLA. The following conclusions can be drawn from the results of this study:

1. Raising the dosage of ARP or TPS decreased the strengths of the biocomposites. The tensile and flexural strengths of the samples containing 0 wt.% ARP, 10 wt.% TPS, and 90 wt.% PLA were 18.73 MPa and 84.28 MPa, respectively, The strengths of the samples containing 11 wt.% ARP, 0 wt.% TPS and 89 wt.% PLA were 23.07 MPa and 105.39 MPa, respectively, while those of the samples containing 11 wt.% ARP, 10 wt.% TPS and 79 wt.% PLA dropped to 17.46 MPa and 82.06 MPa, respectively.
2. All of the composites were more easily thermally decomposed than PLA. Increasing the percentage of TPS or ARP in the biocomposites resulted in the samples becoming more thermally unstable.
3. After immersion in water for 7 days, the masses of all of the samples would be changed due to the dissolvement of TPS and the water absorption by the samples. For sample A containing 90 wt.% PLA and 10 wt.% TPS, the mass change rate was negative. With the increase in the dosage of ARP, the mass change rate of the samples increased gradually to positive values. When the content of ARP was kept constant in the specimens, the mass change rate was monotonically reduced with the increase in the amount of TPS.
4. Soil burial altered the surfaces and fracture surfaces of the samples. After soil burial, the surface color became uneven, some components detached from the samples, leaving some micro holes on the bodies. The fracture surfaces became much rougher, and more cracks or holes appeared with the prolonging of the soil-burial time.
5. Extending the soil-burial time resulted in the samples’ extensive mass loss and reduction in storage modulus and flexural properties. After soil burial for 180 d, the weight, the storage modulus at 30 °C, as well as the flexural strength and modulus, were greatly reduced by 21.40%, 38.36%, 42.02% and 44.19%, respectively, when compared with those before soil burial.
6. Extending soil-burial time increased the thermal stability and decreased the crystallinity of the samples gradually, but had little effect on the glass transition temperature, cold crystallization temperature or melting point of the samples.

Through experimentation, it is confirmed that ARP/TPS/PLA can be subjected to degradation in soil and the incorporation of ARP and TPS can accelerate the degradation of PLA.

## Figures and Tables

**Table 1:** Compositions of ARP/TPS/PLA blends.

Sample ID	A	B	C	D	E
PLA (wt.%)	90	85	79	84	89
TPS (wt.%)	10	10	10	5	0
ARP (wt.%)	0	5	11	11	11

**Table 2:** The parameters for the FDM 3D printing of ARP/TPS/PLA biocomposite samples.

Parameter	Print Temperature/°C	Layer Thickness/mm	Print Speed/(mm/s)	Deposition Angle/°
Data	220	0.1	50	0

**Table 3:** Thermogravimetric analysis information table of TPS/ARP /PLA ternary composites at different formulations.

Sample	Ti/°C	Tp/°C	W/% (550 °C)
A	335.8	370.3	5.92
B	334.1	363.8	5.33
C	326.4	354.9	5.27
D	327.6	356.3	3.68
E	331.3	362.4	3.93

**Table 4:** Thermogravimetric analysis information of different specimens.

Soil-Burial Time/Day	T_i_/°C	T_p_/°C	Char Residue/% (550 °C)
0	329.4	354.8	7.72
30	336.3	363.6	7.28
60	337.5	364.7	7.79
90	338.9	366.8	2.43
180	340.0	367.5	2.72

**Table 5:** DSC thermal information of ARP/TPS/PLA samples at different soil-burial durations.

Soil-Burial Time/d	T_g_/°C	T_cc_/°C	T_m_/°C	ΔH_cc_/(J/g)	ΔH_m_/(J/g)	Χ_c_/%
0	62.7	121.2	150.9	−13.70	16.76	4.2
30	62.2	122.2	151.0	−18.38	19.26	1.2
60	62.0	124.3	151.1	−7.83	8.67	1.1
90	61.9	124.4	151.2	−14.81	14.52	0.4
120	61.7	124.6	151.4	−15.87	16.13	0.3

## Data Availability

Not applicable.
